# Elements of Physiology

**Published:** 1852-01

**Authors:** 


ARTICLE IV.

*Elements of Physiology including Physiological Anatomy.* By William B. Carpenter, M. D., F. R. S., &c. Second American from a new and revised London edition, with one hundred and ninety illustrations; ps. 566. Philadelphia: Blanchard & Lea, 1851. (From the Publishers through S. C. Griggs & Co.)

The general reputation of this work is such that it is only necessary for us to mention its appearance in a new and improved edition to secure it the attention that it deserves. Although we might join issue with the author on several points of physiological doctrine had we time to review them, we must, for the present, let this brief notice suffice.

